# Bronchopleural Fistula Following COVID‐19 and Necrotizing Pneumonia in Childhood: Treatment With Intrapleural Vacuum‐Assisted Closure Therapy

**DOI:** 10.1002/rcr2.70134

**Published:** 2025-03-20

**Authors:** Jose Carlos Fraga, Felipe Colombo Holanda, Natalia Zanini Silva, Maria Fernanda Oliveira, Paola Santis Isolan, Elenara Fonseca Procianoy

**Affiliations:** ^1^ Pediatric Thoracic and Airway Surgery Units, Hospital de Clínicas de Porto Alegre, Department of Surgery, School of Medicine Universidade Federal do Rio Grande do Sul Porto Alegre Brazil; ^2^ Pediatric Thoracic and Airway Surgery Units Hospital de Clínicas de Porto Alegre Porto Alegre Brazil; ^3^ Hospital de Clínicas de Porto Alegre Porto Alegre Brazil; ^4^ Universidade Federal do Rio Grande do Sul Porto Alegre Brazil; ^5^ Pediatric Surgery Service, Hospital de Clinicas of Porto Alegre, Department of Surgery, School of Medicine Federal University of Rio Grande do Sul Porto Alegre Brazil; ^6^ Pediatric Pulmonology Unit Pediatric Service, Hospital de Clínicas de Porto Alegre Porto Alegre Brazil

**Keywords:** bronchopleural fistula, children, COVID‐19 infection, necrotizing pneumonia, vacuum‐assisted closure therapy

## Abstract

Bronchopleural fistula is an abnormal communication between the bronchial tree and the pleural space. Necrotizing pneumonia is a catastrophic infection of the lungs characterised by necrosis of the interstitial tissue. Although rarely reported, it has been described as a complication of COVID‐19. The usual treatment for bronchopleural fistula involves endoscopic or surgical procedures. We present the case of a 5‐year‐old girl admitted with necrotizing pneumonia and difficult‐to‐control bronchopleural fistula, with a report of flu‐like illness and a positive PCR test for COVID‐19 2 months earlier. Over 38 days, several procedures were performed, including left‐side tube drainage, thoracotomy, segmental resection, open window thoracotomy, and finally vacuum‐assisted closure (VAC) therapy. Twenty days after the start of VAC therapy, complete closure of the bronchopleural fistula was achieved. At 26 months of follow‐up, the child remained asymptomatic, with radiological examinations showing pulmonary expansion and absence of intrapleural air.

## Graphical abstract

Report for the first time of the successful use of vacuum‐assisted closure therapy in a child for the treatment of bronchopleural fistula reluctant to other types of treatment.

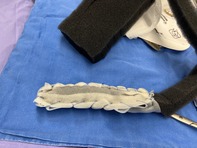

## 1 Introduction

Necrotizing pneumonia (NP) has been reported in individuals with a history of COVID‐19, but only rarely in children [1]. One possible complication of NP is the development of bronchopleural fistulas (BPF), which are associated with significant morbidity and mortality [2]. The procedures of choice for BPF closure include endoscopic or surgical interventions, which may, however, be contraindicated or ineffective. Alternatively, the use of vacuum‐assisted closure (VAC) therapy for the management of BPF has been reported in adults [3] but has not yet been described in children.

## 2 Case Report

**FIGURE 1:**
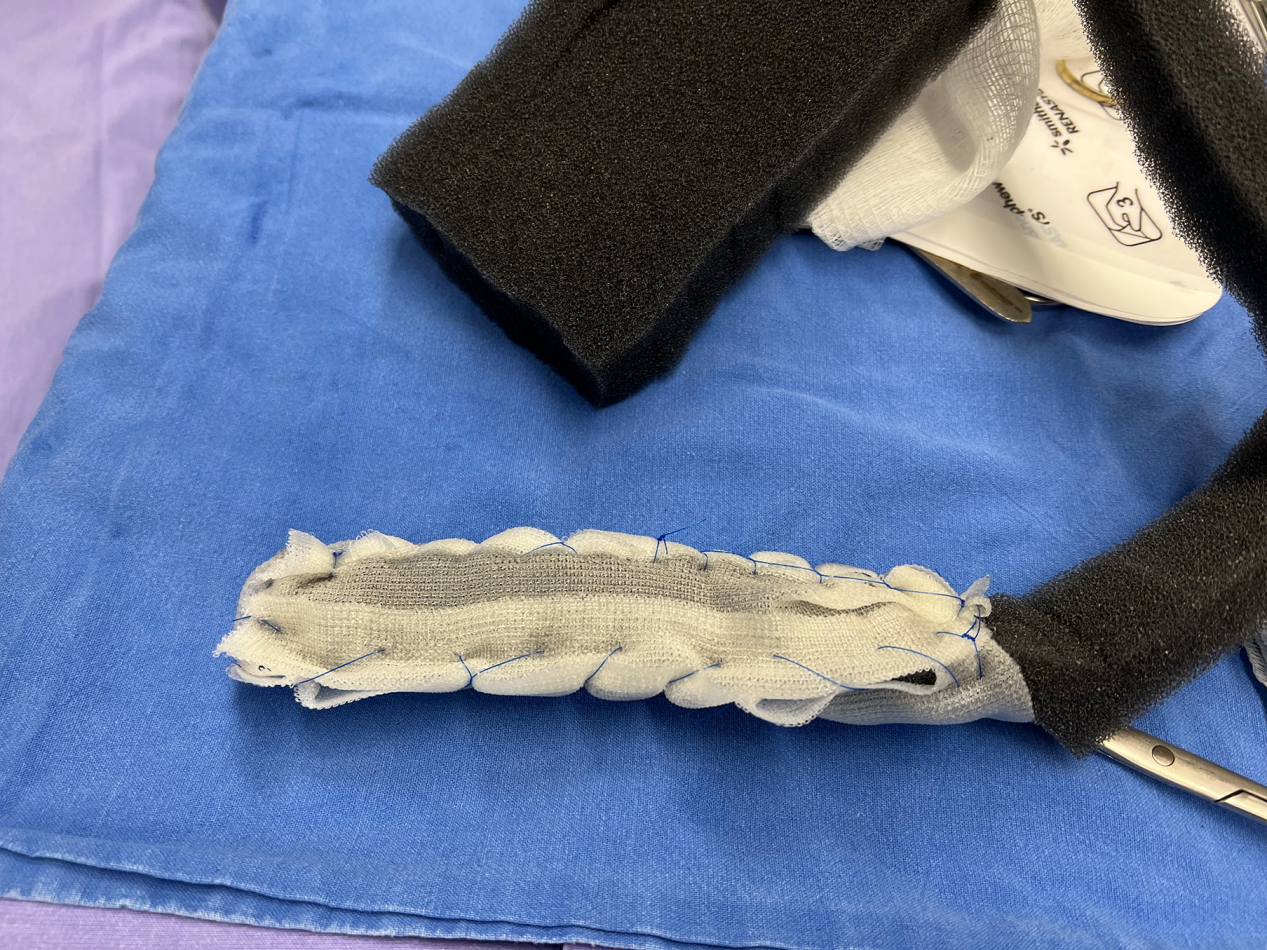
Sponge covered with a non‐adhering dressing (Adaptic) before introduction into the pleural space through OWT.

**FIGURE 2:**
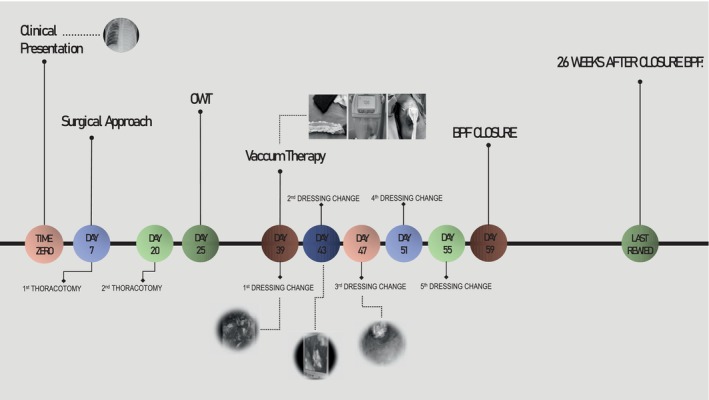
Timeline of BPF closure. OWT, open‐window thoracotomy; BPF, bronchopleural fistula.

A 5‐year‐old girl was admitted with pneumonia and left‐sided hydropneumothorax, with a report of flu‐like illness and a positive PCR test for COVID‐19 2 months earlier. After left‐sided tube drainage, a high‐output BPF was detected, which did not close after 7 days and required surgery. Thoracotomy revealed necrotizing pneumonia (NP) with several perforations in the left upper lobe (LUL). Segmental resection was performed using a stapler and the perforations were sutured. Lung histology showed bacterial pneumonia with vessel thromboembolism and haemorrhagic parenchymal infarction. A chest tube inserted to control air leakage was removed after the leakage ceased 12 days later. Nevertheless, the patient developed progressive ventilatory difficulties, with chest computed tomography (CT) revealing a large pneumothorax and multiple BPFs in the left lung. A second thoracotomy was performed with resection of necrosis in the LUL and suturing of BPFs in the LUL and left lower lobe (LLL). The drain was removed after 3 days, but the patient still developed progressive left chest wall emphysema. Chest x‐ray showed a large pneumothorax cavity in the left upper hemithorax. An open window thoracotomy (OWT) was performed, revealing an incarcerated lung and high‐output BPFs. A VAC system was placed through the OWT, maintaining negative pressures ranging from 25 to 140 mmHg with a vacuum pump (RENASYS Negative Pressure Wound Therapy; Smith & Nephew). Vacuum dressing was changed every 4 days. The sponge was covered with a non‐adhering dressing (Adaptic) before being introduced into the pleural space (Figure 1). Prior to placement of the 2nd dressing, thoracoscopy through the OWT showed three fistulas (Video 1). On the 3rd dressing, one was occluded. CuraCel and Gelfoam were used to close the remaining fistulas. On the 4th dressing, one single BPF was observed. On the 5th dressing, the BPF persisted with a large amount of granulation tissue but significant reduction in pneumothorax size. CuraCel and Gelfoam were replaced. Twenty days after the start of VAC therapy, complete closure of the BPF was observed, with no air leaks even at high ventilatory pressures (Figure 2). CT showed significant pulmonary expansion of the left lung, with a small residual air cavity in the left upper hemithorax, but without evidence of BPF. At 26 months of follow‐up, the patient is asymptomatic, with normal spirometry and chest x‐ray and CT with lung expansion and no air cavities in the left hemithorax.

**VIDEO 1:** Video showing timeline and thoracoscopy images of vacuum dressings. Video content can be viewed at https://onlinelibrary.wiley.com/doi/10.1002/rcr2.70134 Video content can be viewed at https://onlinelibrary.wiley.com/doi/10.1002/rcr2.70134

## 3 Discussion

NP in children is a severe form of lung disease with great morbidity [2]. Although it typically occurs as a complication of community‐acquired pneumonia, our case illustrates that NP may also occur as a secondary infection, even after COVID‐19 pneumonia with a favourable outcome. The presence of haemorrhage and pulmonary thromboembolism in the resected lung confirms the histological changes associated with COVID‐19 infection. In the present case, the lung injury caused by the virus may have favoured the occurrence of pulmonary necrosis and the formation of a difficult‐to‐control BPF. The presence of BPF leads to contamination of the pleural cavity and the contralateral lung with bacteria due to aspiration of infected material [3].

Treatment of BPF involves controlling the infection and closing the air leak with endoscopic or surgical procedures [4]. The placement of sealing substances or valves via bronchoscopy can only be performed in the presence of a single or small BPFs [3]. Surgical treatment involves closing the air leaks with sutures [3]. However, as observed in our case, surgery may not be effective when performed on infected or ischaemic lung parenchyma and may also entail extensive lung resections. In the case of multiple and refractory BPFs, no approach has proven to be as effective as surgical stepwise treatment carried out in two stages, involving OWT followed by spontaneous closure or open drainage followed by a second surgical procedure to accelerate BPF closure [3]. In adults, Pairolero et al. [5] recommend the use of a muscle flap to cover and occlude the BPF in addition to performing open chest drainage. Our case showed that it is not always necessary to cover the BPF with a muscle flap, given that progressive lung expansion may be sufficient to occlude the BPF.

The use of VAC therapy can be beneficial in the treatment of difficult‐to‐control BPF as it speeds up the clearing and healing of the infected area and allows for rapid lung expansion and occlusion of the BPF. VAC creates negative pressure in the pleural cavity, accelerates the formation of granulation tissue, reduces the number of bacteria, and removes excess local fluid, improving oxygenation and reducing the volume of the pleural space [3]. Covering the sponge with a non‐adhering dressing prevents it from sticking to the lung parenchyma and also prevents the removal of local granulation tissue when the dressing is changed. The risk of mediastinal shifts during the application of negative intrapleural pressure by the vacuum dressings is minimal, since the lung is incarcerated and the mediastinum is fixed by the pleural infection.

## Author Contributions

Jose Carlos Fraga, Felipe Colombo Holanda, Natalia Zanini Silva, Maria Fernanda Oliveira, Paola Santis Isolan, and Elenara Fonseca Procianoy conceived and designed the study. Jose Carlos Fraga, Felipe Colombo Holanda, Natalia Zanini Silva, and Elenara Fonseca Procianoy cared for the patient during hospitalisation. Jose Carlos Fraga, Felipe Colombo Holanda, Natalia Zanini Silva, Maria Fernanda Oliveira, Paola Santis Isolan, and Elenara Fonseca Procianoy collected and interpreted all relevant clinical data. Jose Carlos Fraga prepared the manuscript. Jose Carlos Fraga, Felipe Colombo Holanda, Natalia Zanini Silva, Maria Fernanda Oliveira, Paola Santis Isolan, and Elenara Fonseca Procianoy confirm the authenticity of all the raw data in this study. All authors read and approved the final manuscript.

## Ethics Statement

Written informed consent was obtained from the patient/caregiver to include their information in this publication. The Institutional Review Board (IRB) of the Hospital de Clínicas of Porto Alegre approved the study protocol and publication of data (CAAE number 82925324.8.0000.5327).

## Conflicts of Interest

The authors declare no conflicts of interest.

## Data Availability

The data that support the findings of this study are available on request from the corresponding author. The data are not publicly available due to privacy or ethical restrictions.
